# Constructing high-order functional networks based on hypergraph for diagnosis of autism spectrum disorders

**DOI:** 10.3389/fnins.2023.1257982

**Published:** 2023-08-31

**Authors:** Jie Yang, Fang Wang, Zhen Li, Zhen Yang, Xishang Dong, Qinghua Han

**Affiliations:** ^1^Faculty of Nature, Mathematical & Engineering Sciences, King’s College London, London, United Kingdom; ^2^School of Information Science and Engineering, Zaozhuang University, Zaozhuang, China; ^3^Hydrological Center of Zaozhuang, Zaozhuang, China; ^4^School of Artificial Intelligence, Zaozhuang University, Zaozhuang, China

**Keywords:** high-order functional connectivity network, resting-state functional magnetic resonance imaging (rs-fMRI), hypergraph, autism spectrum disorder (ASD), classification fusion

## Abstract

**Introduction:**

High-order functional connectivity networks (FCNs) that reflect the connection relationships among multiple brain regions have become important tools for exploring the deep workings of the brain and revealing the mechanisms of brain diseases. The traditional high-order FCN constructed based on the “correlation of correlations” strategy, is a representative method for conducting whole-brain connectivity analysis and revealing global network characteristics. However, whole-brain connectivity analysis may be affected by noise carried by less important brain regions, resulting in redundant information and affecting the accuracy and reliability of the analysis. Moreover, this type of analysis has a high computational complexity.

**Methods:**

To address these issues, a new method for constructing high-order FCN based on hypergraphs is proposed in this article, which is used to accurately capture the real interaction relationships among brain regions. Specifically, first, a low-order FCN reflecting the connection relationships between pairs of brain regions based on resting-state functional Magnetic Resonance Imaging (rs-fMRI) time series is constructed, the method first constructs the low-order FCN that reflects the connection relationships between pairs of brain regions based on rs-fMRI time series, and then selects the “good friends” of each brain region from hypergraph perspective, which refers to the local friend circles with closer relationships. Then, the rs-fMRI time series corresponding to the “good friends” in each brain region’s friend circle are averaged to obtain a sequence that reflects the intimacy between brain regions in each friend circle. Finally, hypergraph high-order FCN, which reflects the interaction relationships among multiple brain regions, is obtained by calculating the correlations based on the sequence of friend circles.

**Results:**

The experimental results demonstrate that the proposed method outperforms traditional high-order FCN construction methods. Furthermore, integrating the high-order FCN constructed based on hypergraphs and the low-order FCN through feature fusion to achieve complementary information improves the accuracy of assisting in the diagnosis of brain diseases.

**Discussion:**

In addition, the effectiveness of our method has only been validated in the diagnosis of ASD. For future work, we plan to extend this method to other brain connectivity patterns.

## 1. Introduction

Functional connectivity networks (FCNs) based on resting-state functional magnetic resonance imaging (rs-fMRI) (Liu et al., 2008; Smith et al., 2013) reflect the connectivity between pairs of brain regions and provide a measure of temporal correlations in brain activity. However, the brain is a complex and highly efficient network, and FCNs only capture low-order interactions between brain regions, ignoring the complex high-order relationships among multiple brain regions. In order to comprehensively reflect the complex interaction patterns among multiple brain regions, some researchers have used a strategy based on “correlation of correlations” to construct high-order FCNs, and to explore deep-level functional connectivity interaction information (Plis et al., 2014; Chen et al., 2017; Guo et al., 2017; Zhou et al., 2018).

For example, Zhao et al. (2018) used a “correlation of correlations” strategy to construct multi-level high-order FCNs based on rs-fMRI data for diagnosing ASD, achieving a classification accuracy of 81%. Zhang et al. (2016) used second-order correlations based on Pearson’s correlation (Pc) to reflect the complex high-order relationships among brain regions, and this method has a high sensitivity in detecting inter-group differences between normal individuals and patients. Yu et al. (2014) used Pc to investigate a multilevel high-order FCN based on low-order FCN for the diagnosis of ASD. The “correlation of correlations” strategy involves performing two consecutive correlation calculations on the rs-fMRI time series. Although these traditional high-order FCNs are effective in identifying and classifying neurological diseases (Jia et al., 2017; Zhao et al., 2018), they consider all the connections among brain regions, which is a whole-brain connectivity analysis, which may lead to two issues. (1) It may result in noise from unimportant brain regions, affecting the accuracy and reliability of the analysis results. (2) Since a large number of connections among brain regions need to be computed, there may be information redundancy, long computation time, and high computational complexity. Therefore, it is worth exploring how to efficiently, quickly, and accurately identify brain regions closely related to the recognition of brain diseases.

**Figure 1:**
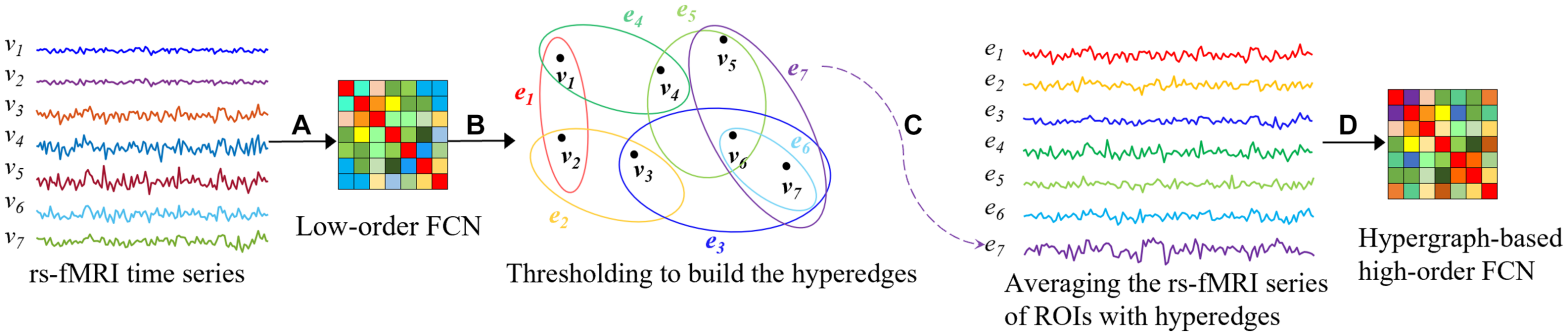
Construction of hypergraph-based high-order FCN. $v_{i}$ : the rs-fMRI time series that reflects the changes in the blood oxygen signal of the i -th brain region over a period of time, $e_{i}$ : the hyperedge in the hypergraph (1≤i≤7) . **(A)** Constructing an FCN based on rs-fMRI time series; **(B)** Constructing hyperedges based on the concept of hypergraphs; **(C)** Averageing the rs-fMRI series of ROIs with hyperedges; **(D)** Hypergraph-based high-order FCN.

Hypergraph is a tool that can well describe the association relationships in complex systems, and it is widely used in brain network analysis in neuroscience (Yu et al., 2014). Compared with other methods, hypergraphs can not only represent high-order relationships among multiple vertices, but also better distinguish the importance of different vertices and edges, leading to a more accurate functional analysis of brain networks. With these advantages of hypergraphs, a novel method of constructing high-order FCNs based on hypergraph is proposed to achieve a more realistic and accurate capture of the connection relationships among brain regions in this paper. We illustrate the construction process of the proposed method using seven brain regions as an example in Figure 1. First, a low-order FCN that reflects the connectivity between pairs of brain regions is constructed based on the rs-fMRI time series, as shown step (A) in Figure 1, where $v_{i}$ represents the rs-fMRI time series that reflects the changes in the blood oxygen signal of the i -th brain region over a period of time. Next, as shown step (B) in Figure 1, from a hypergraph perspective, the “good friends” of each brain region are selected based on low-order FCN, which are the local communities of brain regions that have closer connectivity relationships, where $e_{i}$ represents the hyperedge in the hypergraph, reflecting the connections between closely related brain regions. Then, the rs-fMRI time series corresponding to each brain region and its “good friends” are normalized to obtain a sequence reflecting the intimacy level of the community, as shown step (C) in Figure 1. Finally, hypergraphs of high-order brain networks reflecting the interaction relationships between multiple brain regions are obtained by computing correlations based on sequences of community, as shown step (D) in Figure 1. The hypergraph-based high-order FCN construction method can overcome the noise problems caused by high computational complexity, information redundancy, and insufficiently tight connectivity relationships of brain regions in whole-brain connectivity analysis.

**Figure 2:**
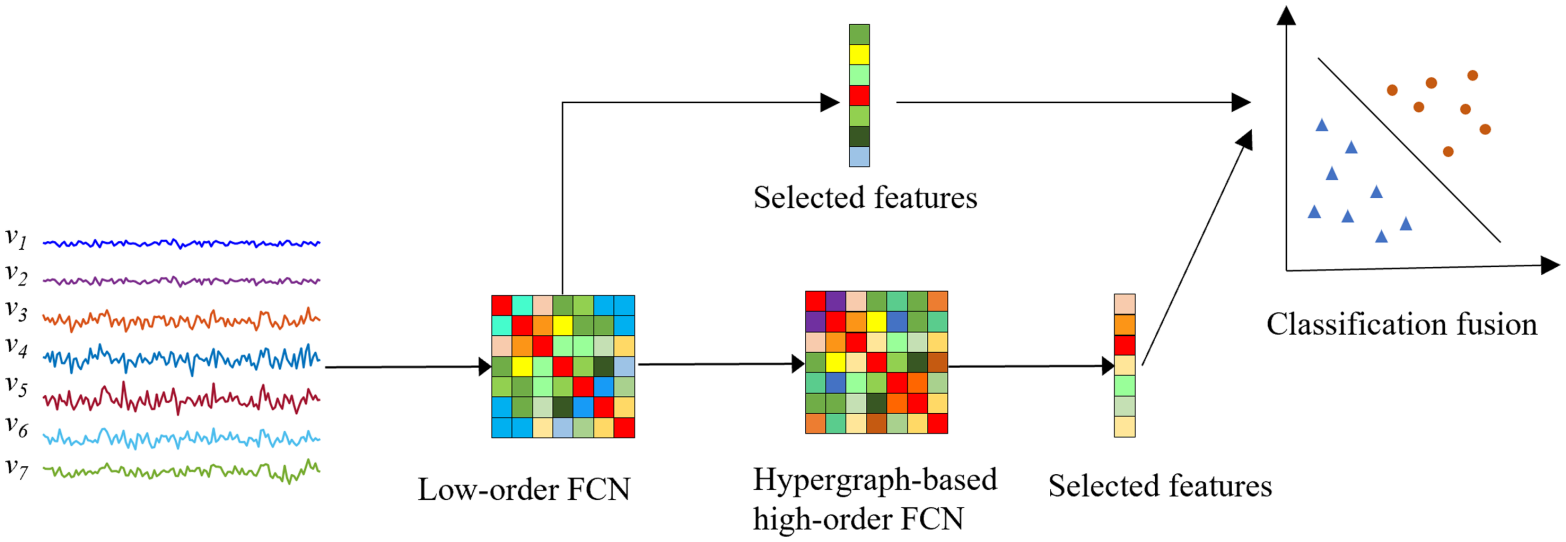
Overview of the proposed fusion framework for ASD diagnosis. $v_{i}$ : the rs-fMRI time series that reflects the changes in the blood oxygen signal of the i -th brain region over a period of time (1≤i≤7) .

Overall, as shown in Figure 2, the pipeline of the proposed classification framework in this paper mainly includes the following four steps: (1) Construction of low-order FCNs. We first construct low-order FCNs reflecting the connectivity between pairs of brain regions based on the original rs-fMRI time series. Each low-order FCN is represented as a correlation matrix. (2) Construction of hypergraph-based high-order FCNs. Traditional high-order FCNs are constructed from a global perspective based on the “correlation of correlations” strategy, while hypergraph-based high-order FCNs are constructed from a hypergraph perspective. (3) Feature selection based on two-sample t-test and least absolute shrinkage and selection operator (LASSO). We use the elements in the high-order FCNs obtained in step (2) and the elements in the low-order FCNs obtained in step (1) as features for each individual, and then perform feature selection to select the most relevant features for the classification task. (4) Classification fusion. We first use two linear support vector machines (SVMs) to construct an ensemble classifier, then train the classifier with the features obtained in step (3), and finally produce the final classification result by weighted averaging the SVM classification scores.

The main contributions of our method are as follows: (1) Compared with traditional high-order FCNs, using hypergraph theory to construct high-order FCNs can not only reduce computational complexity but also reduce information redundancy and noise, which is conducive to improving the accuracy of brain region analysis. (2) The fusion of traditional low-order FCNs and hypergraph-based high-order FCNs can achieve complementary feature advantages, resulting in improved diagnostic performance for brain diseases.

The organization of this paper is as follows. In Section 2, we describe the preparation of the data, the methods related to the conventional high-order FCN construction, and our proposed high-order FCN construction. In Section 3, we report the experimental setup and evaluate the effectiveness of our proposed method through its application to identification tasks. Furthermore, we investigate the effect of different thresholds on the proposed high-order FCN constructions, and the most discriminative super-edge connections. Finally, the full paper is concluded in Section 4.

## 2. Materials and methods

### 2.1. Data acquisitions and processing

This study utilized resting-state functional magnetic resonance imaging (rs-fMRI) data from the Autism Brain Imaging Data Exchange (ABIDE) database, which includes 539 individuals with ASD and 573 normal control (NC) subjects from 17 international imaging centers (Di Martino et al., 2014). The detailed scan procedures and protocols are described on the ABIDE website^1^. Considering that several sites contain only a limited number of participants, we use data from 5 different sites, including NYU, Leuven, UCLA, UM and USM. Specifically, rs-fMRI scan data from 45 individuals with ASD and 47 typically developing controls were selected from the NUY site. Detailed demographic information is summarized in Table 1.

**Table 1:** Demographic information of the studied subjects from five imaging sites in the ABIDE database.

Site	ASD		NC	
	Age	M/F	Age	M/F
NYU	11.1 ± 2.3	36/9	11.0 ± 2.3	36/11
Leuven	13.10±4.79	21/4	18.80±9.00	24/8
UCLA	16.27±6.48	28/8	14.65±4.79	31/7
UM	17.05±8.36	43/5	17.35±7.12	56/9
USM	15.77±7.21	30/8	17.34±9.53	21/1

The values are denoted as mean ± standard deviation. M, male; F, female.

The data acquisition and preprocessing in this study follow a standard pipeline that include head movement correction, normalization, denoising, and other related processes and parameters, similar to previous literature (Satterthwaite et al., 2013; Yan et al., 2013; Washington et al., 2014; Lin et al., 2015; Ray et al., 2015; Urbain et al., 2016; Reinhart and Nguyen, 2019). Subsequently, the brain was segmented into 116 ROIs using the automatic anatomical labeling (AAL) map, and the mean value of the rs-fMRI time series for each ROI was calculated, generating the data matrix $X\in R^{170\times 116}$ for further analysis. It is important to note that the data matrix consists of 170 time points and 116 brain ROIs.

### 2.2. Functional connectivity network estimation

#### 2.2.1. Baseline method

In this study, we define $x_{i}\in R^{M}$ as the mean rs-fMRI time series computed from all blood-oxygen-level dependent (BOLD) time-series signals corresponding to the voxels within the i -th ROI. Here, *M* denotes the total number of temporal image volumes. For convenience, we will abbreviate the calculation of Pc-based functional connectivity between the i -th and j -th ROIs as:

(1) $$c_{ij}=\mathrm{corr}\left(x_{i},x_{j}\right)\;\;$$

Subsequently, a low-order FCN is generated using conventional Pc-based methods, represented by a symmetric matrix $C_{LON}$ , which is defined as follows:

(2) $$C_{LON}={\left(c_{ij}\right)}_{1\leq i,j\leq M}$$

In the matrix $C_{LON}$ , each row or column represents the Pearson correlation series between a particular ROI and all other ROIs. Every element of $C_{LON}$ is derived from the Pc between the mean time-series of two ROIs, i and j . It is important to note that $C_{LON}$ captures low-order interactions between any pair of ROIs.

**Figure 3:**
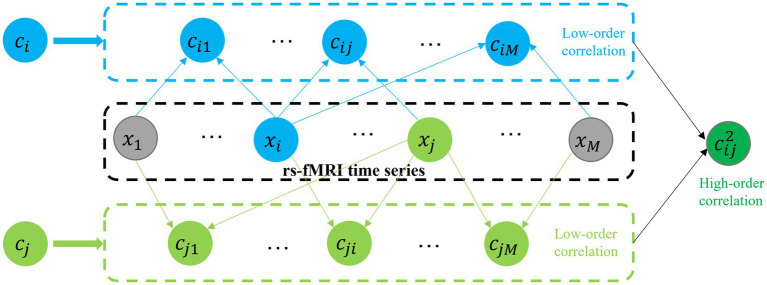
Construction of high-order FCN based on “correlation’s correlation.” $x_{i}$ : the mean rs-fMRI time series computed from all BOLD time-series signals corresponding to the voxels within the i -th ROI. $c_{i}$ : denote the correlations between the i -th ROI and all other ROIs. $c_{ij}^{2}$ : The Pc between $c_{i}\;$ and $c_{j}$ .

In order to capture high-order functional interactions among brain regions, we employ a method proposed in the study (Zhang et al., 2017) to generate high-order FCN based on “correlation’s correlation,” as shown in Figure 3. Specifically, we use a vector $c_{i}=\left(c_{i1},c_{i2},\cdots ,c_{iM}\right)$ to denote the correlations between the i -th ROI and all other ROIs. Mathematically, $c_{i}$ represents the i -th row or column of the symmetric matrix $C_{LON}$ in Equation (2). The “correlation’s correlation” between the i -th and j -th ROIs is computed as follows:

(3) $$c_{ij}^{2}=\mathrm{corr}\left(c_{i},c_{j}\right)$$

where $c_{i}=\left(c_{i1},\cdots ,c_{i\left(i-1\right)},c_{i\left(i+1\right)},\cdots ,c_{i\left(j-1\right)},c_{i\left(j+1\right)},\cdots ,c_{iM}\right)$ and $c_{j}=\left(c_{j1},\cdots ,c_{j\left(i-1\right)},c_{j\left(i+1\right)},\cdots ,c_{j\left(j-1\right)},c_{j\left(j+1\right)},\cdots ,c_{iM}\right).$ The “correlation’s correlation” coefficient, denoted as $c_{ij}^{2}$ , provides insight into how the FCN profiles between the i -th ROI and all other ROIs resemble those between the *j*-th ROI and all other ROIs. This measure reveals more complex relationships among the FCN profiles (or the vectors $\left\{c_{i}\right\}$ ), extending beyond the information captured by the original rs-fMRI time series $x_{i}$ . Consequently, the high-order correlation coefficient $c_{ij}^{2}$ in Equation (3) is capable of extracting interaction information from all ROIs, in contrast to the correlation coefficient $c_{ij}$ in Equation (1), which only involves the two specific ROIs. In other words, $c_{ij}^{2}$ characterizes more complex and abstract interactions among multiple brain regions. Thus, the corresponding high-order matrix can be defined as follows:

(4) $$C_{HON}={\left(c_{ij}^{2}\right)}_{1\leq i,j\leq M}$$

Although $C_{HON}$ is widely used as an important high-order FCN, the high-order correlation coefficient $c_{ij}^{2}$ contains the interaction among all ROIs. In fact, the interaction between some ROIs is weak or even no relationship, using all ROIs information to construct the high-order FCN leads to the redundancy of the matrix and the introduction of noise, which further affects the identification performance.

#### 2.2.2. The proposed method

Simple graphs have been widely used to model relations between two vertices, where each edge connects two vertices and the weight of each edge denoting a specific type of relation between them. However, in many applications, the relationships among the data may be more complex than pairwise connections or second-order relations. In order to effectively capture high-order relationships among multiple vertices and avoid the loss of valuable information that cannot be represented by simple graphs, hypergraphs (Corsini and Leoreanu, 2003) have been developed as a generalization of simple graphs. In hypergraphs, hyperedges can connect any number of vertices, forming non-empty subsets of vertices. To further elaborate, we provide a brief introduction to the basic notations of hypergraphs (Zhou et al., 2006) below.

A hypergraph G=(V,E,w ) is defined by a vertex set $V=\left\{v_{1},v_{2},\cdots ,v_{N}\right\}$ , a hyperedge set $E=\left\{e_{1},e_{2},\cdots ,e_{M}\right\}$ with $\cup_{i=1}^{M}e_{i}=V$ , and a hyperedge weight vector w= ${\left(w\left(e_{1}\right),w\left(e_{2}\right),\cdots ,w\left(e_{M}\right)\right)}^{T}\in R^{M}$ , where each hyperedge $e_{i}$ is assigned a weight $w\left(e_{i}\right)$ for 1≤i≤M. The $C_{HON}$ contains redundant connections in unrelated brain regions. Based on hypergraph theory, a threshold is set for the low-order FCN, denoted by t . The ROI connection pairs above the threshold are retained, and hyperedges are constructed to connect all the retained connection pairs. Thus, the structure of the hypergraph G based on threshold selection can be represented by a matrix $H=\left[H_{ij}\right]\in R^{M\times M}$ with entries defined as follows:

(5) $$H_{ij}=\{\begin{array}{ll} 1, & \;\mathrm{if}\;v_{i}\in e_{j}\\ 0, & \;\mathrm{otherwise}. \end{array}$$

Where $e_{j}$ is defined as the hyperedge that j -th ROIs is connected to other ROIs. $v_{i}$ represents the i -th ROIs that are strongly correlated with j -th ROIs based on threshold t . When $v_{i}\in e_{j}$ , i.e., $H_{ij}=1$ , a hyperedge $e_{j}$ is said to be incident with a vertex $v_{i}$ .Further, averaging the rs-fMRI time series of the ROIs connected by the hyperedges, the averaged rs-fMRI time series obtained for each hyperedge is defined as $x_{i}^{\prime }\in R^{M}$ , i.e., hyperedge series. The Pearson correlation coefficient between the i -th and j -th hyperedges series is calculated using the following Equation (6). Subsequently, hypergraph-based high-order FCN is generated, represented by the symmetric matrix ***H-C***_***HON***_, which is defined in Equation (7).

(6) $$c_{ij}^{\prime }=\mathrm{corr}\;\left(x_{i}^{\prime },x_{j}^{\prime }\right)$$

(7) $$H-C_{HON}={\left(c_{ij}^{\prime }\;\right)}_{1\leq i,j\leq M}$$

In the matrix ***H-C***_***HON***_, each row or column represents the Pearson correlation series between a particular hyperedge and all other hyperedges. Each element of ***H-C***_***HON***_ is derived from the Pc between the average time series for ROIs of two hyperedges ( $e_{i}$ and $e_{j}$ ). Notably, ***C***_***HON***_ elaborates the complexity and abstract interaction information of multiple brain regions but contains a large number of redundant connections and noise between brain regions. In contrast to ***C***_***HON***_, ***H-C***_***HON***_ focuses only on the relationships among some highly connected brain regions, reducing the large number of redundant relationships between brain regions.

### 2.3. FCN feature extraction, selection and classification

For the l -th subject, we use its corresponding low-order FCN matrices ***C***_***LON***_ and ***H-C***_***HON***_ as raw features. Since the ***C***_***LON***_ and ***H-C***_***HON***_ matrices are symmetric, including duplicate features would result in redundancy. Therefore, we only vectorize their lower off-diagonal triangular part to define the feature vectors, i.e., $y_{0}^{\left(l\right)},y_{1}^{\left(l\right)}$ , for representing the l -th subject’s ***C***_***LON***_ and ***H-C***_***HON***_, respectively. The dimensionality of $y_{i}^{\left(l\right)}$ is $\frac{M\left(M-1\right)}{2}$ , where M denotes the number of ROIs for $y_{0}^{\left(l\right)}$ , and the number of hyperedges for $y_{1}^{\left(l\right)}$ .

The feature vectors $y_{0}^{\left(l\right)},y_{1}^{\left(l\right)}$ extracted from ***C***_***LON***_ and ***H-C***_***HON***_ might include redundant or irrelevant features for ASD diagnosis. Thus, feature selection is necessary. For selecting a small subset of features most relevant to the pathology of ASD, we use the two-stage feature selection strategy. Step 1: Performing a two-sample *t*-test between normal controls (NCs) and ASD subjects for each feature in $y_{0}^{\left(l\right)},y_{1}^{\left(l\right)}$ . Those features whose *p*-values are smaller than a certain threshold are retained. At this point, we label the newly obtained feature set $y\prime_{0}^{\left(l\right)},y\prime_{1}^{\left(l\right)}$ . Step 2: Adopting $L_{1}$ -norm regularized least squares regression, known as LASSO (Tibshirani, 1996), due to its efficiency and simplicity (Jin et al., 2015; Jie et al., 2016; Wee et al., 2016). Specifically, let $\theta_{j}={\left(w_{i1},w_{iz},\cdots ,w_{id}\right)}^{T}$ represent the weight vector for the feature selection task and $K={\left(k_{1},k_{2},\ldots ,k_{N}\right)}^{T}$ are the class labels of N training data (from N training subjects). Here, d is the number of features. Mathematically, the LASSO model can be described as follows:

(8) $$\frac{1}{2}\overset{N}{\underset{i=1}{\sum }}\;{\left\|\left.k_{l}-{\left(y_{i}^{\left(l\right)}\right)}^{T}\omega_{i}\right\|\right.}_{2}^{2}+\lambda {\left\|\omega_{i}\right\|}_{1}$$

where λ is a parameter for controlling the strength of $L_{1}$ norm regularization. The first term in Equation (8) is the empirical loss on the training data, and the second term is the $L_{1}$ norm regularization term that is used to enforce some elements of $\omega_{1}$ to be zero (i.e., corresponding to non-discriminative features in our classification task). In this way, we can jointly achieve classification error minimization and sparse feature selection. Let ${\tilde{y}}_{0}^{\left(i\right)},{\tilde{y}}_{1}^{\left(i\right)}$ denote selected features from the original feature vectors in the first stage $y\prime_{0}^{\left(l\right)},y\prime_{1}^{\left(l\right)}$ .

After selecting the most important features by LASSO, we use SVM with a linear kernel for ASD classification (Cortes and Vapnik, 1995). SVM aims to find a hyperplane with the maximum margin to effectively separate the samples of one class from another.

### 2.4. FCN evaluation

To assess the performance of the H-C_HON_, we train classifiers for ASD diagnosis using the conventional C_LON_, H-C_HON_ and their fusion (FUSION), respectively. It is worth noting that FUSION is fused by a linear combination of C_LON_ and H-C_HON_. Specifically, the two SVMs are trained using C_LON_ and H-C_HON_, and the output of each SVM is used as the classification result. And then the final classification results are obtained by fusing the decision scores of all SVMs. Since the combination coefficients are difficult to determine in practical applications, they are simply fused by 0.5 × (C_LON_ + H-C_HON_) in this paper.

In this experiment, we adopt a nested tenfold cross-validation strategy consisting of two nested loops to evaluate the classification performance. The outer loop involves dividing the 92 subjects into 10 subsets of comparable size, where one subset is designated as the test set, and the other nine subsets are used as the training set. In the inner loop, the train set is combined and redistributed into 10 subsets of similar size, with nine subsets used for tuning the hyperparameters and one for model evaluation. Our method’s performance is primarily affected by three hyperparameters: *p* and λ in feature selection and γ in the SVM model. The optimal hyperparameters are determined when the average classification accuracy reaches its maximum. We determine the optimal values for the parameters in the following ranges: p∈[0.01:0.01:0.1] , λ∈[0.1:0.1:0.9] and $\gamma \in \left[2^{-4},\cdots ,2^{4}\right].$ Once the optimal hyperparameters are selected in the inner loop, they are returned to the outer loop where the model is trained on the training dataset and evaluated on the test set.

## 3. Experimental analyses

### 3.1. Classification performance

In our experiments, we adopt six metrics to evaluate different FC construction methods: classification accuracy (ACC), sensitivity or true positive rate (TPR), specificity or true negative rate (TNR), positive predictive value (PPV), negative predictive value (NPV), and F1 score. Denote TP, TN, FP and FN as True Positive, True Negative, False Positive, and False Negative, respectively. Those evaluation metrics can be defined as follows: ACC = (TP + TN)/(TP + TN + FP + FN), SEN = TP/(TP + FN), SPE = TN/(TN + FP), BAC = (SEN + SPE)/2, PPV = TP/(TP + FP), and NPV = TN/(TN + FN). For these metrics, higher values indicate better classification performance. In addition, we performed the statistical significance test (*t*-test) on the accuracy obtained by three comparison methods and FUSION, and the value of ps of the test are also listed in Table 2. When the value of p is less than 0.05, it indicates that there is a significant difference between the two methods.

**Table 2:** Demographic information of the subjects.

Target site	Method	ACC (%)	SEN (%)	SPE (%)	PPV (%)	NPV (%)	*p*-Values	F1 (%)
	C_LON_	73.81	77.00	71.00	77.25	81.87	0.012	73.24
NUY	C_HON_	74.06	76.00	72.00	76.05	78.98	0.017	73.66
	H-C_HON_	79.81	80.00	86.50	88.50	82.79	0.021	79.05
	Fusion	**80.31**	**83.00**	**87.12**	**89.48**	**86.12**	/	**80.11**
	C_LON_	75.13	79.06	64.05	66.31	65.84	0.023	72.62
Leuven	C_HON_	76.54	79.67	65.83	68.13	68.91	0.031	75.72
	H-C_HON_	80.27	79.78	73.62	76.25	73.48	0.038	74.95
	Fusion	**82.94**	**80.51**	**78.35**	**79.42**	**82.16**	/	**79.93**
	C_LON_	78.64	79.87	77.54	78.27	80.69	0.029	72.43
UCLA	C_HON_	79.71	80.09	78.67	79.24	79.31	0.021	73.87
	H-C_HON_	83.45	83.11	81.21	81.31	81.61	0.018	76.51
	Fusion	**85.98**	**83.86**	**83.58**	**84.26**	**83.53**	/	**79.25**
	C_LON_	70.09	67.05	68.51	70.49	73.41	0.027	74.00
UM	C_HON_	71.86	71.39	72.67	72.01	75.72	0.015	74.86
	H-C_HON_	75.21	75.26	77.37	75.65	76.64	0.011	78.15
	Fusion	**78.36**	**77.43**	**78.16**	**79.51**	**78.88**	/	**79.96**
	C_LON_	77.23	73.41	72.00	74.13	73.54	0.043	71.79
USM	C_HON_	78.68	74.61	73.27	75.24	74.16	0.035	72.91
	H-C_HON_	82.01	77.84	78.71	76.69	77.92	0.016	77.24
	Fusion	**85.02**	**81.96**	**82.01**	**80.31**	**79.36**	/	**79.69**

To demonstrate the robustness of the test results, we conducted experiments on the real multi-site ASD dataset with five imaging sites (NYU, Leuven, UCLA, UM, and USM). The experimental results are shown in Table 2. The results from each site consistently indicate that the proposed high-order FCN (H-C_HON_) outperforms compared to the two baseline methods, C_LON_ and C_HON_. The best performance is highlighted in bold. It is worth noting that for the experiments conducted on C_LON_ and C_HON_, no free parameters were involved. For the proposed method, we set the threshold to 0.7, which yielded the best performance in ASD identification.

Based on the experimental results shown in Table 2, we can draw the following conclusions: (1) The proposed high-order FCN has better performance than the conventional high-order FCN, indicating that constructing a high-order FCN using the idea of hypergraphs likely reduces redundant information and related noise, thereby improving the accuracy of brain analysis. Additionally, setting a threshold allows for the elimination of weak connections between brain regions, which helps improve computational efficiency and reduce complexity. (2) The fusion of H-C_HON_ and C_LON_ is better than any single FCN, suggesting that different levels of brain networks contain distinct features. Feature fusion potentially enables the integration of complementary information, enhancing the comprehensiveness of discriminative features and facilitating the identification of brain disorders such as ASD.

### 3.2. The influence of parameters on H-C_HON_

In general, the selection of free parameters in FCN construction methods plays a crucial role in determining the final classification performance. In the proposed method, we investigate the influence of the threshold t that constitutes the set of vertices of the hyperedges on the performance of C_HON_ using data from the NUY site.

To evaluate the sensitivity of our method to t , we repeat the identification experiments based on threshold steps [0.1:0.05:0.95], and discuss the effect of different thresholds on the final classification performance. Table 3 reports the individual evaluation metrics for different thresholds, and the best results are shown in bold. From Table 3, we find that the choice of threshold is crucial to the classification performance, and different thresholds determine different network topologies, which can provide different useful information for ASD identification and obtain different classification performance. We observe that the proposed method exhibits the highest performance when the threshold is set to 0.7 across all evaluation metrics.

**Table 3:** Classification performance corresponding to different threshold parameters.

Regularization parameter	ACC (%)	SEN (%)	SPE (%)	PPV (%)	NPV (%)	F1 (%)
0.1	66.22	60.50	71.50	70.17	68.56	61.50
0.15	65.69	62.50	68.50	67.33	66.14	63.44
0.2	68.81	59.00	78.00	72.17	69.10	63.38
0.25	71.31	65.50	77.00	79.50	70.14	68.83
0.3	73.56	69.50	77.50	79.50	74.87	71.54
0.35	73.19	71.50	75.00	76.71	75.14	72.55
0.4	70.31	68.00	73.00	67.17	73.75	66.28
0.45	71.44	71.50	72.00	72.67	72.83	71.13
0.5	74.56	69.50	80.00	78.83	74.00	72.33
0.55	78.42	80.00	77.50	80.50	80.58	77.98
0.6	75.56	78.00	73.50	74.33	79.50	75.27
0.65	79.06	80.00	78.50	80.00	82.67	78.37
0.7	**79.81**	**80.00**	**86.50**	**88.50**	**82.79**	**79.05**
0.75	79.17	72.00	80.00	81.81	79.35	76.14
0.8	78.06	74.00	82.50	83.67	78.95	76.28
0.85	78.81	78.00	80.00	80.67	81.48	77.81
0.9	74.56	74.00	75.50	76.67	77.79	73.36
0.95	74.81	72.00	78.00	78.64	78.14	72.60

From Table 3, we can conclude two conclusions: (1) When the threshold is less than the optimal threshold 0.7, the H-C_HON_ lacks specific expressiveness and loses much relevant information, which is not conducive to the auxiliary diagnosis of ASD and other brain disorders. (2) When the threshold is larger than the optimal threshold of 0.7, it represents that most of the connections among brain regions are preserved. Whereas, the lower diagnostic accuracy of brain diseases suggests that the information representation of the H-C_HON_ may be redundant and contain a large amount of non-essential noise-related information. Therefore, choosing an appropriate threshold setting is crucial to improve the performance of the proposed higher-order FCN.

### 3.3. The most discriminative hyperedge connection ASD diagnosis

To identify the most discriminative features in the H-C_HON_, we utilized two-sample t-test and LASSO. In this study, we quantify the correlation between features and target classification by using the frequency of feature selection across all cross-validation cases.

The H-C_HON_ with the highest frequency in tenfold cross-validation was selected as the most discriminative connection. The reported results were based on the original Automated Anatomical Labeling (AAL) atlas, which comprises 116 brain regions (Tzourio-Mazoyer et al., 2002). In this section, we only analyze the H-C_HON_ with the best classification accuracy threshold at 0.7. The five most discriminative hyperedge connections identified were hyperedge $e_{1}$ and $e_{18}$ , hyperedge $e_{1}$ and $e_{48}$ , hyperedge $e_{1}$ and $e_{56}$ , hyperedge $e_{1}$ and $e_{94}$ , hyperedge $e_{2}$ and hyperedge $e_{67}$ . Since one hyperedge represents multiple connections for two groups of ROIs, we trace back to the connections between the two groups of ROIs based on the hyperedge connections.

**Figure 4:**
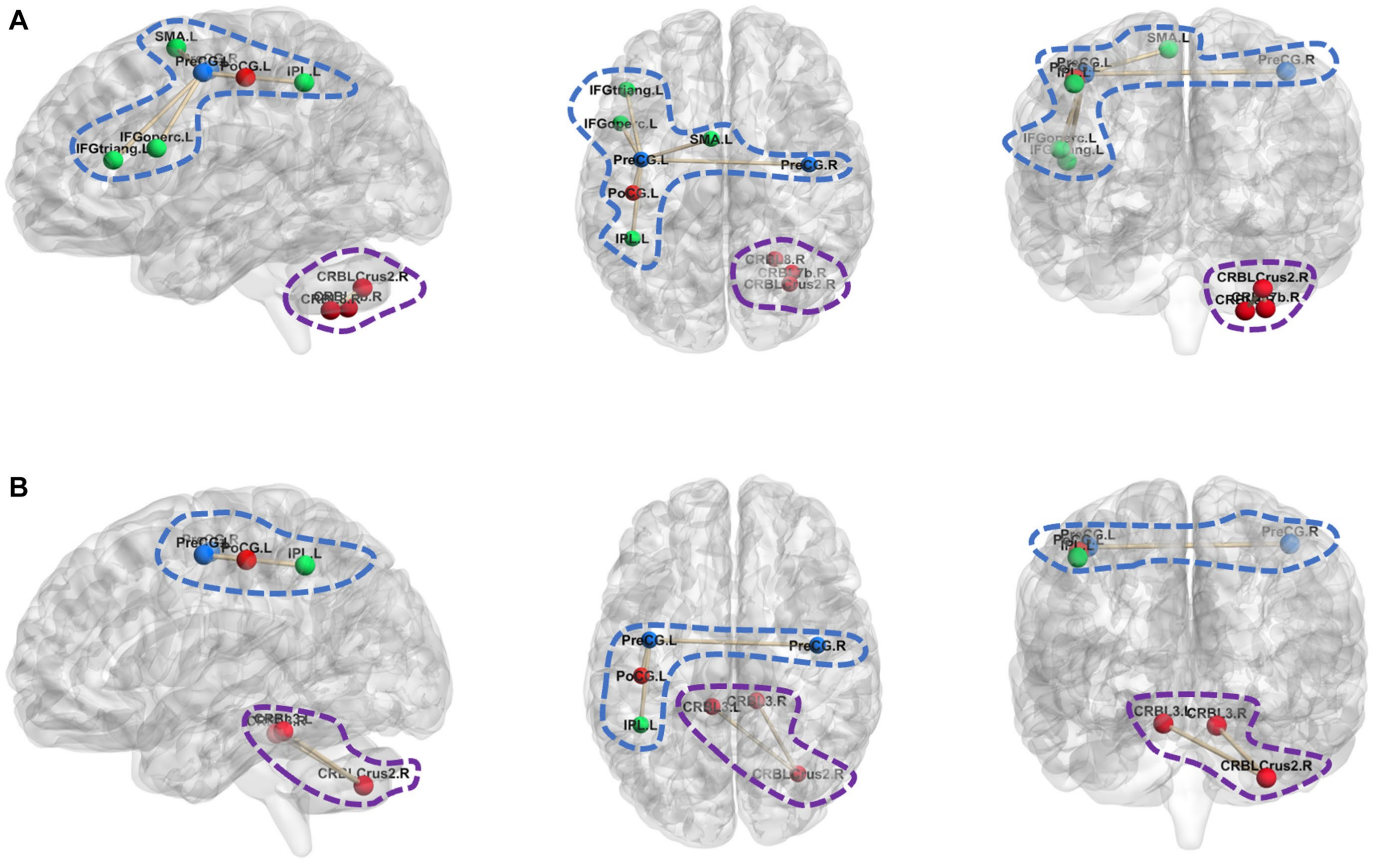
ROIs differences in the most discriminative hyperedge connection. **(A)** The ROIs connected by hyperedge e1 and hyperedge e94 in ASD; **(B)** The ROIs connected by hyperedge e1 and hyperedge e94 in NC.

Figure 4 shows the most discriminative hyperedge connections in five sets of hyperedge connections, which were the hyperedges connected by hyperedge $e_{1}$ and hyperedge $e_{94}$ . Figures 4A,B respectively represent the ROIs connected by hyperedge $e_{1}$ and hyperedge $e_{94}$ in ASD and NC, where the areas surrounded by blue dashed lines and purple dashed lines are the ROIs connected by hyperedge $e_{1}$ and hyperedge $e_{94}$ respectively. As shown in Figure 4, there are obvious differences in the ROIs connected by hyperedges in the most discriminative hyperedge connections of ASD and NC.

Hyperedge $e_{1}$ connects several brain regions centered around left Precentral gyrus (PreCG.L). In NC, $e_{1}$ connects to PreCG. L, right Precentral gyrus (PreCG.R), left Postcentral gyrus (PoCG.L), and left Inferior parietal (IPL.L). In ASD, $e_{1}$ connects to PreCG. L, PreCG.R, PoCG. L, IPL.L, left supplementary motor area (SMA.L), left Inferior frontal gyrus (opercular) (IFGoperc.L) and left Inferior frontal gyrus (triangular) (IFGtriang.L). By comparing $e_{1}$ in NC and ASD, it is suggested that there may be abnormal connections between PreCG. L and SMA. L, PreCG.L and IFGoperc. L, as well as PreCG. L and IFGtriang.L in individuals with ASD. Nebel et al. (2014) found that the strength of connectivity within and between distinct functional subregions of the PreCG was related to ASD diagnosis and to the severity of ASD traits. Zhang et al. (2020) found differences in convergence in the SMA.L when comparing the NC sample with the ASD sample. IFGoperc.L and IFGtriang.L belong to the Inferior frontal gyrus. Rudie et al. (2012) indicate that the inferior frontal gyrus, especially its posterior portion, has an important role in imitation and social reciprocity and in the pathophysiology of their disturbance in ASD. Hyperedge $e_{2}$ connects several brain regions centered around right Cerebellar Crus 2 (CRBL Crus 2.R). In NC, $e_{2}$ connects to right CRBL Crus 2, left Cerebellar3 and right Cerebellar3. In ASD, $e_{2}$ connects to CRBL Crus 2.R, right Cerebellar8 and right Cerebellar7b. It can be observed that there are abnormalities in the Cerebellar of between NC and ASD. Kelly et al. (2021) that cerebellar dysfunction is increasingly associated with ASD.

## 4. Discussion

There are several limitations in this paper. Firstly, while multi-site data increases sample size and statistical power, it introduces site heterogeneity. To address this issue, learning shared features across multiple sites becomes particularly important to mitigate data heterogeneity. Secondly, we ignored the spatiotemporal dynamic interactions between brain regions at different time points. In fact, the spatial interactions between brain regions at the previous time point can affect the spatial interactions between brain regions at the next time point. To address this issue, we plan to incorporate the hypergraph concept and attention mechanism to capture the spatiotemporal dynamic features of the brain graph network. Finally, the effectiveness of our method has only been validated in the diagnosis of ASD. For future work, we plan to extend this method to other brain connectivity patterns.

In addition, to further advance the field, future research can focus on the following aspects: Firstly, exploring advanced feature extraction and selection techniques to enhance the discriminative power of high-order FCNs. Secondly, conducting comparative studies with other state-of-the-art methods on multiple datasets to gain a more comprehensive understanding of the strengths and weaknesses of different approaches. Finally, investigating the interpretability of the models and providing insights into underlying brain mechanisms can greatly facilitate the applicability of the proposed method in clinical settings.

## 5. Conclusion

In this paper, we propose a novel hypergraph-based high-order FCN, which constructs high-order FCN by averaging multiple related ROI connected by hyperedges, and the integration of conventional low-order FCN (C_LON_) and hypergraph-based high-order FCN (H-C_HON_) to improve classification performance. The method is characterized by its simplicity and effectiveness, as it can capture high-order connectivity patterns between brain regions and reduce redundancy in high-order FCN. Experimental results showed that C_LON_ and H-C_HON_ have certain complementarity and combining them effectively can improve classification accuracy. And the proposed H-C_HON_ achieves a classification accuracy of 80.31% by combining with C_LON_ through SVM fusion. At present, this study only explores the construction of static high-order FCN, and in the future, we plan to extend the hypergraph-based method to the construction of dynamic high-order networks.

## Data availability statement

Publicly available datasets were analyzed in this study. This data can be found at: http://fcon_1000.projects.nitrc.org/indi/abide/abide_l.html.

## Author contributions

JY: Conceptualization, Formal analysis, Investigation, Methodology, Software, Validation, Writing – original draft. FW: Conceptualization, Methodology, Writing – review & editing. ZL: Writing – review & editing. ZY: Writing – review & editing. XD: Writing – review & editing. QH: Writing – review & editing.

## Funding

The author(s) declare financial support was received for the research, authorship, and/or publication of this article.

This work was supported by University-Industry Innovation Fund of China (Nos. 2022BL097 and 2021ITA09023), and Natural Science Foundation of Shandong Province under Grant ZR2017LF010.

## Conflict of interest

The authors declare that the research was conducted in the absence of any commercial or financial relationships that could be construed as a potential conflict of interest.

## Publisher’s note

All claims expressed in this article are solely those of the authors and do not necessarily represent those of their affiliated organizations, or those of the publisher, the editors and the reviewers. Any product that may be evaluated in this article, or claim that may be made by its manufacturer, is not guaranteed or endorsed by the publisher.

## Footnotes

^1^ http://fcon_1000.projects.nitrc.org/indi/abide/abide_I.html
